# Using the NASSS–Complexity Assessment Tool to Evaluate the Implementation of “Cadê O Kauê?”: Chat-Story Intervention for Youth Participation in Mental Health Promotion in Brazil

**DOI:** 10.2196/79106

**Published:** 2026-05-27

**Authors:** Sakshi Setia, Gabriela Pavarini, Mona Koshkouei, Sheila Giardini Murta

**Affiliations:** 1 Nuffield Department of Primary Care Health Sciences, University of Oxford, Radcliffe Primary Care Building, Radcliffe Observatory Quarter, Woodstock Rd, Oxford, OX2 6GG, United Kingdom; 2 Department of Social Policy and Intervention, University of Oxford, 32 Wellington Square, Oxford, OX1 2ER, United Kingdom, 44 1865 270325; 3 Department of Clinical Pyschology, Universidade de Brasília, Brasilia, Brazil

**Keywords:** digital mental health, implementation science, mental health, knowledge translation, youth participation

## Abstract

**Background:**

The global public health challenge of young people’s mental health is particularly evident in Brazil, which is considered the most anxious and the fifth most depressed country globally. In response to this, a digital intervention, that takes the form of a storytelling chatbot named “Cadê o Kauê?” (translation: “Where is Kauê?”), was coproduced by an interdisciplinary team of researchers, young people, and narrative designers as part of a project titled Engajadamente. The intervention was designed to strengthen Brazilian adolescents’ skills in promoting their peers’ mental health through direct support and collective action for mental health. The intervention was rolled out in select schools in Brazil and online via social media, but it encountered challenges during the initial implementation phase in school settings.

**Objective:**

This project’s primary objective is to evaluate the implementation of “Cadê o Kauê” in school settings in Brazil, assess current and potential complexities, and formulate recommendations to reduce or manage these complexities. The secondary objective of this project is to understand the current and potential sociotechnical change resulting from the implementation of the “Cadê o Kauê” in a real-world setting.

**Methods:**

The Non-adoption, Abandonment, Scale-up, Spread, Sustainability (NASSS) framework was selected as the theoretical framework to identify different areas of complexity across domains and further thinking of ways to reduce or manage these complexities. Data were gathered by conducting semistructured online interviews with the Engajadamente team members based in the United Kingdom and Brazil. The NASSS–Complexity Assessment Tool was used as a sensemaking device to facilitate a collective understanding of the implementation, and narratives were mapped onto specific NASSS domains. Key themes were identified across overarching domains using thematic analysis to meet the objectives of this evaluation.

**Results:**

The results mapped onto the NASSS domains generated a narrative about the initial implementation of “Cadê o Kauê?” in schools. Existing and potential complexities across technology, organization, and wider system domains were characterized by interdependencies, unintended consequences, and uncertainties. The implementation of “Cadê o Kauê?” highlighted challenges related to internet dependency, infrastructure limitations, and varying levels of organizational readiness, teacher preparedness, and school culture. Addressing these through offline solutions, additional teacher training, whole-school engagement strategies, and a value-informed complexity approach can enhance accessibility, alignment with school priorities, and adaptability within Brazil’s dynamic sociopolitical context.

**Conclusions:**

Using the NASSS framework, the evaluation captured both current and emerging complexities in implementing “Cadê o Kauê?” in schools. By engaging with theory-informed approaches to technology implementation projects and deep-diving into discursive dynamic interactions between technology and its “context” of implementation, we have explored ways to manage these complexities and developed suitable recommendations to guide the Engadajamente team and support similar projects in the future.

## Introduction

Mental ill health represents the leading cause of disability among individuals aged 10‐24 years, accounting for 45% of the global burden of disease [1,2], where half of mental health disorders begin by the age of 14 years [3]. The global public health challenge of young people’s mental health is particularly evident in Brazil, which is considered the most anxious and fifth most depressed country globally [4]. The COVID-19 pandemic severely impacted Brazil; around 1 in 3 young people exhibited symptoms of depression or anxiety [5].

In low- and middle-income countries (LMICs), sociocultural factors, including religion, cultural values, economic inequality, and political instability, disproportionately impact mental health outcomes [2,6,7]. For instance, Brazilian adolescents from lower socioeconomic backgrounds face greater mental health challenges and poor access to care [8]. Determinants such as stigma surrounding mental health also affect care-seeking and mental health service usage across the country [9]. Novel solutions are required to address these sociocultural determinants for promoting young people’s mental health in Brazil and other LMICs [10].

Since the United Nations Conventions on Rights of the Child (1989), researchers, practitioners, and policymakers have increasingly acknowledged young people’s potential to be active agents of social change capable of promoting health in their communities through participation in advisory groups, representative councils, or activism [11]. Grounded in the Ottawa Charter’s definition of health promotion (1986), mental health promotion reflects a view of health as a positive concept encompassing social and personal resources, as well as physical capacities. Accordingly, youth participation in promoting mental health within the community involves engaging in social interactions and collective action to address social determinants and to build community capacities and competencies for well-being, rather than focusing solely on illness and its risk factors [11].

Since adolescents spend a significant amount of their time in school, schools have become natural environments for promoting positive mental health outcomes [12-14]. School initiatives focusing on mental health promotion have strengthened students’ preparedness to offer peer support and a sense of belonging within the school community [15].

Over the past decade, digital interventions, such as mobile apps, have been recognized as essential tools for addressing young people’s mental health needs by overcoming traditional barriers and expanding access to care, particularly in underserved communities [16]. In parallel, co-designing these interventions with young people provides scalable and culturally relevant solutions to support their mental health [17]. However, most co-designed digital mental health interventions have been developed and evaluated in high-income regions, including Australasia, North America, and Europe [18]. This highlights an important gap, as there is a pressing need for contextually relevant digital mental health resources in LMICs, where health care access remains limited, and stigma surrounding mental health persists [19,20].

“Cadê o Kauê?,” is one such digital mental health intervention, which takes the form of a storytelling chatbot, developed to enhance young people’s participation in promoting mental health and well-being within their peer communities in Brazil [21]. This approach reflects a potentially scalable intervention model that builds on peer networks, strengthens community connections, and encourages youth engagement in collective action and social advocacy [22]. It was co-designed by *Engajadamente’s* [22] interdisciplinary team of researchers, adolescents, and narrative designers to strengthen Brazilian adolescents’ skills in promoting their peers’ mental health through direct support and collective action for mental health [23]. The development also involved input from teachers, psychologists, and other high school professionals through advisory committees [24]. The detailed auto-ethnography of the pluralistic coproduction model [21,23] is summarized in another paper.

In this digital narrative, players interact with fictional, automated characters (bots) via SMS text messaging and learn that their friend is struggling with his mental health. As the narrative progresses, they “unlock” skills (eg, signposting and establishing partnerships) that enable them to support him and others [24]. The chat-story was initially envisioned for remote, stand-alone use among adolescents aged 15‐18 years. However, following the mapping stage of the co-design process, the tool was tailored for use within schools to create a more comprehensive pathway for change, with teachers responsible for facilitating the introduction of “Cadê o Kauê?” in classrooms with students [21]. A feasibility trial accompanied by a qualitative acceptability study of “Cadê o Kauê?” was conducted in 7 schools [21]. Several unanticipated challenges emerged during the initial implementation in school settings, indicating the necessity of further real-world evaluation of “Cadê o Kauê?”

Implementing large-scale technology-supported projects does not simply involve introducing new technology or disrupting old practices but also navigating an intricate web of interconnected factors within those systems [25]. This requires a mindset grounded in complexity thinking, recognizing these systems’ dynamic and unpredictable nature [26]. Complexity, as defined by Cohn et al [27], is a “dynamic and constantly emerging set of processes that not only interact with each other but come to be determined by those interactions.” Effective implementation, therefore, necessitates viewing technological interventions as part of a larger social system, where success depends on embracing the system’s inherent complexity [26]. Complex adaptive systems have fuzzy boundaries that allow for dynamic interactions between components of the system, leading to emergent behaviors. Undertaking sociotechnical evaluations of complex technology projects offers an opportunity to understand the changing and evolving interrelationships between technology and human or organizational (or socio-) factors [28]. A sociotechnical perspective assumes that “organizational and human (socio) factors and technology’s (technical) materiality are interrelated parts of one system, recursively (not simultaneously) shaping each other” [29,30].

Previous research has evaluated the feasibility and acceptability of “Cadê o Kauê?” through user surveys as part of the co-design process [21]. Survey findings indicated that the intervention enhanced adolescents’ motivation and self-efficacy to engage in peer support and collective action related to mental health, as well as increasing their openness in discussing mental health issues [21].

However, to understand “how” “Cadê o Kauê?” functions within the broader social and institutional context of implementation, this qualitative study uses a complexity science approach for evaluation of this intervention. The primary aim of this study is to evaluate the implementation of “Cadê o Kauê?” to identify existing and potential complexities and develop recommendations to address or mitigate these challenges. The secondary aim is to explore potential sociotechnical changes associated with implementing “Cadê o Kauê?” in a real-world context.

## Methods

### Theoretical Framework

**Figure 1.**
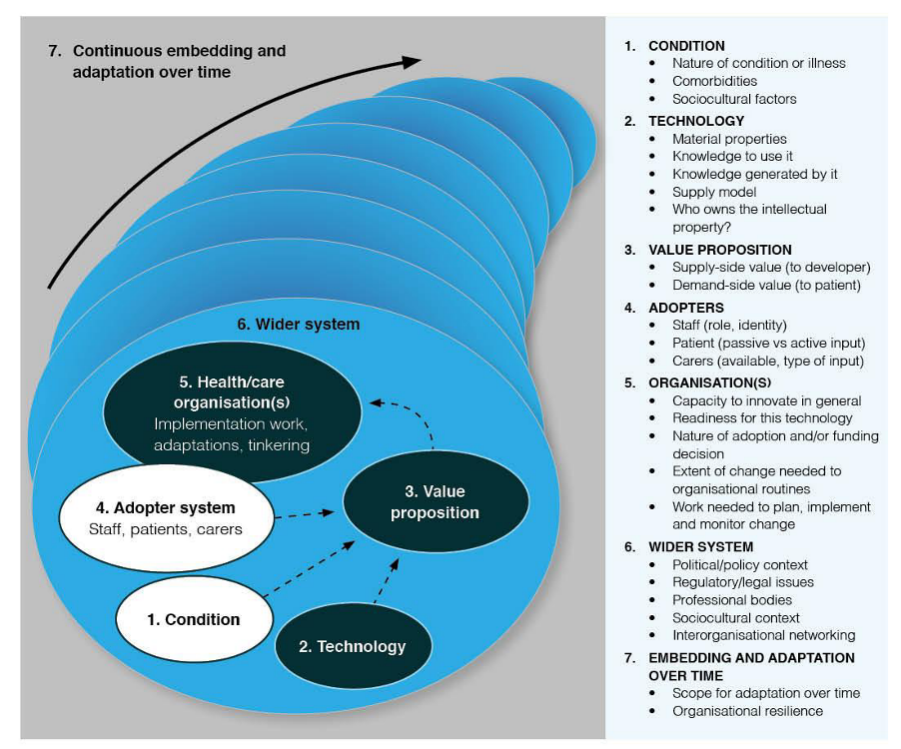
The NASSS framework (reproduced from Greenhalgh et al [31]) published under the Creative Commons Attribution 4.0 International License [32]. NASSS: Non-adoption, Abandonment, Scale-up, Spread, Sustainability.

The Non-adoption, Abandonment, and Challenges to Scale-up, Spread, and Sustainability (NASSS) framework is selected as the theoretical framework to gain insights into the multifaceted and interdependent components of the initial implementation of “Cadê o Kauê?.” This framework was developed against the backdrop of complex adaptive systems, synthesizing various theories to map possible areas of complexity when planning, evaluating, and synthesizing a technology initiative in health and social care [25]. NASSS framework (Figure 1) consists of 7 domains—the condition or illness, the technology, the value proposition, the adopter system (intended users), the organizations, and the wider system (mainly regulatory, legal, and policy issues); the seventh, cross-cutting, domain considers how all these interact and emerge over time [24].

NASSS is used for this study on the account of the following: first, “Cadê o Kauê?” was introduced into a complex landscape with overlapping system boundaries involving young people, teachers, school administration, and wider policy structures. NASSS is well-suited to address cultural, infrastructural, and policy-specific complexities by addressing interactions between the technology and the implementing organization and the wider social system [25]. Second, the framework supports the use of retrospective exploration of “how” and “why” “Cadê o Kauê?” functioned as it did during early implementation, rather than focusing solely on whether the intervention was effective. This is particularly important for identifying emergent interactions, contextual influences, and unintended consequences that were not fully visible at the outset of implementation. Third, it has been previously used to evaluate digital mental health interventions, including those in LMICs [33].

### Participants and Procedure

With the help of a senior researcher (GP), we identified a purposive sample of stakeholders with varied roles and involvement in the co-design and implementation of the intervention to capture their diverse perspectives. This sample included core co-design team members, including researchers, youth collaborators, technology and policy experts, and implementation facilitators, including 2 teachers from schools that tested out the intervention (Table 1). Stakeholders from the United Kingdom and Brazil (Table 1) were invited via personalized emails, after which they reviewed a participant information sheet and gave informed consent through Microsoft Forms. Participant Information Sheets and consent forms were provided in English and Portuguese, depending on participants’ preferences.

**Table 1.** Summary of study participants’ characteristics.

Participant ID	Location of the participant	Role	Mode of data collection	Language	Role in Engajadamente project
Participant ID 1	Brazil	Youth collaborator	Synchronous	English	Member of core co-design team
Participant ID 2	United Kingdom	Researcher	Synchronous	English	Member of core co-design team
Participant ID 3	United Kingdom	Researcher	Synchronous	English	Member of core co-design team
Participant ID 4	Brazil	Researcher	Synchronous	English	Member of core co-design team
Participant ID 5	Brazil	Researcher	Synchronous	English	Member of core co-design team
Participant ID 6	Brazil	Policy expert	Synchronous	Portuguese	Member of advisory committee advising on policy aspects
Participant ID 7	Brazil	Tech expert	Synchronous	Portuguese	Member of core co-design team
Participant ID 8	Brazil	Teacher	Asynchronous	Portuguese	Testing in schools
Participant ID 9	Brazil	Teacher	Asynchronous	Portuguese	Testing in schools
Participant ID 10	Brazil	Youth collaborator	Asynchronous	Portuguese	Member of core team for co-design process

### Data Collection

Data collection took place in 2024 virtually with support from Microsoft Teams. Interviews were conducted virtually due to the geographical dispersion of participants across different countries and time zones, as well as their varied professional commitments. Online interviews provided a flexible and feasible approach that enabled participation without the need for travel, while still allowing for in-depth discussion. Virtual platforms also supported secure audio recording and note-taking, and did not compromise the quality of interaction. In total, 7 interviews took place synchronously and lasted for an average of 56 minutes (43‐60 min). Furthermore, 3 interviews (2 teachers and 1 youth collaborator) were conducted asynchronously, with the questions answered through audio messages. This alternate strategy was used to facilitate participation, as teachers had limited time to participate in the study and were, therefore, difficult to recruit.

A semistructured interview guide (Multimedia Appendix 1) was designed for online interviews using the prompts from the theoretical NASSS framework, covering 7 domains—condition, technology, value proposition, adopters, organization, wider system, and emergence over time. For online interviews, questions were divided into three sections, that are (1) participant’s background, Brazilian context, and technology; (2) questions about the implementation process in schools, including the success factors and challenges faced; and (3) reflections and future recommendations. Questions were adapted according to participants’ background and their role in the implementation. For example, the interview guide was adapted to cover the technical and material components for the tech-expert, aligning with the prompts from the technology domain of the NASSS framework. For teachers, questionnaires were adapted to fit the relevant domains (organization and adopter system) of the framework. Similarly, for the policy expert to understand the broader context, the guide included detailed follow-up questions on educational and social policies on youth mental health in Brazil. To mitigate recall bias, as interviews were conducted retrospectively, participants were prompted to anchor their reflections to specific implementation milestones, and accounts were interpreted alongside other participant perspectives and available project documentation.

Out of 7 synchronous interviews, 5 were conducted in English, and 2 of them were conducted in Portuguese. All 3 asynchronous interviews were conducted in Portuguese. Interviews were conducted by SS, and GP helped facilitate the conversations for non-English speaking interviewees. The interviews were transcribed using Microsoft Teams transcription software. The transcriptions in Portuguese were translated into English thereafter and checked for accuracy with the help of a native Portuguese-speaking researcher.

To support participant well-being during interviews, questions focused on their experiential learnings of the implementation of the tool, which posed minimal risk. Interviews were conducted by SS, who had previous experience in facilitating qualitative interviews requiring sensitive communication. Participants were reminded that they may pause, decline to answer any question, or stop the interview at any time. All data were handled confidentially, with identifying details removed during transcription and reporting. These measures ensured that risks remain minimal while respecting the sensitive context of youth mental health–related work.

### Ethical Considerations

The Oxford Tropical Research Ethics Committee reviewed the study’s protocol. It was determined that the project did not require further ethical approval in the United Kingdom as it qualifies as a service evaluation project. In Brazil, local ethics approval was sought and granted by the University of Brasilia Humanities and Social Sciences Ethics Committee (reference 6.980.535).

This study focuses on professionals’ and team members’ experiences of implementation rather than their personal mental health; however, the authors acknowledge the broader sensitivity of youth mental health work and co-design contexts. Several safeguards were therefore put together in place. Informed consent was obtained before participation via a Microsoft Form, supported by a detailed participant information sheet (Multimedia Appendix 2). This clearly explained the study purpose, the voluntary nature of involvement and participation, the right to withdraw at any time, and how data will be stored, anonymized, and used. Contact details for the researcher and the University’s ethics or information compliance teams were also provided.

### Data Availability

The University of Oxford is the data controller with respect to participants’ personal data and determined how those data were used. The University processed personal data for the purpose of the evaluation outlined above. Interview recordings were stored securely on the University drives, with access restricted to the study team, and were deleted after data analysis was complete. Consent records were also stored securely on the University drives with restricted access and will be retained for at least 3 years following final publication of the work. Following transcription, any identifying information relating to participants or their organizations was removed, and transcripts were labeled using random IDs. These transcripts may be uploaded to data repositories or shared with other researchers via OneDrive, where appropriate, and were retained for at least 3 years. A separate file linking participant IDs to names and contact details was maintained in case recontact was required and was deleted after data analysis was completed.

### Data Analysis

Data analysis was led by the first author (SS), at the time of the study, an MSc researcher in Translational Health Sciences with experience in qualitative research. Qualitative data from interview transcripts were analyzed using the approach to thematic analysis by Braun and Clarke [34]. Data analysis was conducted simultaneously with data collection, allowing for themes identified during data analysis to inform data collection. In line with this approach, analysis was recursive rather than strictly linear, with ongoing engagement with emerging patterns throughout the period of data collection. Early analytic insights informed areas of attention in subsequent interviews [34]. To understand “the depth and breadth of data” [35], SS familiarized herself with the data by reading and rereading the transcripts multiple times. As a nonnative Portuguese speaker, SS remained attentive to potential linguistic and cultural nuances during interpretation. This was addressed through consultation with bilingual members of the research team to ensure conceptual clarity and contextual accuracy in the analysis. Her year-long engagement with the *Engajadamente* [22] team also enriched her awareness of the pertinent sociopolitical factors surrounding the project. Furthermore, initial interpretations of the data were discussed in meetings with GP and MK, both senior researchers. Personal biases and assumptions that may have been held while analyzing the content were recognized and discussed during those meetings. These sense-checking discussions supported contextual validity while maintaining analytic independence. After the first 5 interviews, the transcripts were imported into NVivo software (Lumivero) [36] to support the data analysis.

Following data familiarization, a coding framework was developed using NASSS as a sensitizing device while keeping the initial evaluation objectives in mind. However, this alone did not define the scope of the findings. Subthemes were identified using NASSS domains as overarching main themes. The coding and subtheme generation process evolved throughout the data analysis process; this process was refined iteratively, with modifications made to the coding framework to capture newly arising concepts and subthemes. After 8 interviews, no substantially new insights were generated across NASSS domains and 2 additional interviews supported the development of a sufficiently rich and coherent set of themes. Subthemes were generated not solely based on the number of codes they contained but also on their significance and how well they related to the evaluation objectives. Earlier transcripts were checked again after data collection was complete to ascertain if new codes could be applied to these transcripts. Any codes that did not fit the overall analysis were checked to see if they could be merged with another code. In the second cycle of analysis, codes were organized into subthemes under the 7 broader domains of the NASSS framework (refer to Results section).

## Results

### Mapping Complexity Onto NASSS Domains

Thematic analysis of interview transcripts was mapped onto the NASSS domains (Table 2). Within each domain, subthemes were identified and illustrated with participant quotes (referenced by participant ID). These subthemes were further interpreted to generate insights, which then informed a detailed examination of complexity within the relevant domains, as presented in the subsequent section.

**Table 2.** Summary of NASSS^a^ domains with corresponding subthemes and supporting participant quotes.

NASSS domain and subtheme	Example data	Insights generated
Condition		
Capturing diverse perspectives of young people’s mental health across Brazil	*“The tool needed to be culturally relevant and sensitive to the diverse realities of Brazilian adolescents. A poor adolescent may have a different set of responsibilities, a Black adolescent faces others, and an adolescent from a middle-class background will have different ones. However, they are all united by the shared adolescent experience.”* [Participant ID 10]	Brazil’s youth mental health landscape is influenced by several sociocultural determinants however the Engajadamente team managed to incorporate plurality of experiences into the design of “Cadê o Kauê?”
Challenges in incorporating the voices of Indigenous populations	*“We wanted to include the experiences of Indigenous people in Brazil into the design of “Cadê o Kauê?”, but were mindful of the need to have appropriate resources and support in places; especially those in remote or rural areas.”* [Participant ID 3]	Additional resources are required to incorporate the perspectives of Indigenous populations
Technology		
Technical integration and platform dependencies	*“A significant challenge was our reliance on platform updates from services like Messenger and Telegram, as any changes to their code affected our development.”* [Participant ID 7]	Technology has complex technical integrations and is dependent on multiple platforms for functioning
Technology’s functionality is dependent on the internet	*“We provided 3G routers to make up for inconsistent internet access, but the signal needed to be improved, especially when large groups of students were using it simultaneously.”* [Participant ID 2]	Technology’s smooth functioning is dependent on stable internet connectivity
Knowledge generated after introduction of technology	*“There was also some resistance from teachers who were concerned about students accessing social media.”* [Participant ID 5]	Some teachers expressed resistance especially those who held negative beliefs about the use of social media in schools
Knowledge generated after introduction of technology	*“We observed that young people place significant value on internet access, especially when students do not have it but want to check social media on their phones.”* [Participant ID 2]	This dependency highlighted the importance of internet access and the growing use of social media among young people
Innovative nature of technology	*“We decided to use the Hero’s Journey framework, which is commonly used in novel writing, for structuring the story. Since our target audience is young people, we incorporated gamification elements to make the story engaging and relevant.”* [Participant ID 3]	The chatbot incorporated an innovative and interactive design, using storytelling elements grounded in principles of positive psychology
Value proposition		
Added value to the school system	*“The primary objective is to spark dialog and empower students to identify and address the mental health needs within their school.”* [Participant ID 4]	“Cadê o Kauê?” can act as a catalyst in initiating meaningful dialogue surrounding mental health and well-being within the school community
Added value to the school system	*“The tool facilitated important conversations about mental health in schools and among adolescents and teachers, breaking down stigmas and enabling a more open dialog. The project encouraged male students to talk about their feelings and interpersonal relationships and provide support to classmates who cried during classes*.” [Participant ID 9]	By fostering conversations about mental health, the intervention engaged adolescents, teachers, and parents, contributing to stigma reduction and promoting values, such as empathy and social responsibility
Added value to the school system	*“The administration, coordinators, and other teachers viewed this project as a valuable addition given the limited support for students experiencing symptoms of anxiety and depression.”* [Participant ID 8]	Within the school system, the intervention was perceived as adding value, especially after pandemic, when schools struggled to respond adequately to the behavioral and mental health needs of students
Improving access to universal mental health services	“*The toolkit informs students about free, state-provided resources beyond the support available in schools or from parents, including psychologists and social workers who should be present in Brazilian schools. The intervention encourages students to reach out to these professionals*.” [Participant ID 1]	The intervention provides young people with a toolkit highlighting available mental health support resources, mainly through Brazil’s universal health system, that offers mental health services for youth in most states
High costs of maintaining the tool	*“Maintaining a presence on Instagram is very expensive because you have to pay for Meta. If the tool were to be standalone, the maintenance cost is low. However, if we want to integrate it into popular platforms like Instagram and Facebook where young people are active, the cost increases significantly.”* [Participant ID 4]	The sustainment of “Cadê o Kauê?” depends on external funding, which is contingent on demonstrating value on the demand side by delivering benefits to the direct users (young people and their peers) and the broader system, including schools, policymakers, and the healthcare system
Adopter system		
High acceptability	*“Comments like ‘It felt close to my life,’ ‘It represented my experiences,’ and ‘I love that character,’ ‘that character is me’ were particularly touching.”* [Participant ID 2]	The representation in the tool was particularly appreciated, students felt a personal connection to the tool, stating how its characters and scenarios reflected their own experiences
Co-design enhanced user engagement	*“The pluralistic co-production model went beyond mere participation, as we shared power and responsibilities among the senior members of the team, this immersive and iterative approach helped understand and adapt “Cadê o Kauê?” to the sociocultural specificities of the population,”* [Participant ID 1]	The co-design process was attributed as a success factor for the high acceptability of “Cadê o Kauê?” among young people .
Organization		
Assessing organization-innovation fit	*“Expanded coordination with school managers is very important as the leadership guides the sustainment of these opportunities. The administrators must see value in prioritizing these projects; otherwise, they will not put all their efforts into making that project happen.”* [Participant ID 6]	Involvement of school administrators in the coproduction process is required to deliver a streamlined intervention in schools
Teachers’ preparedness and need for additional training	*“Teachers expressed concerns about managing difficult conversations when students raised mental health issues.”* [Participant ID 3] *“In a few schools, students felt that teachers needed more time to be ready to handle the discussion circles.”* [Participant ID 5]	Teachers highlighted the need for additional training to facilitate postintervention discussion circles and address student mental health concerns
Shifting organizational routines	*“Ideally, incorporating the tool and conversation circle into the curriculum should mean no extra work for teachers, as the lesson would already be prepared, however during implementation, the whole process took longer than anticipated.”* [Participant ID 2]	The intervention was designed to be used in a classroom setting in high school and expected to last an hour and a half, which corresponded to approximately 2 class periods
Wider system		
Uncertainty in sociopolitical environment	*“Brazil faced significant challenges over the last few years, including economic decline, political instability, and a major shift towards extreme right-wing policies. The political climate created uncertainty around government acceptance of educational interventions, especially concerning adolescent mental health.”* [Participant ID 5]	The project’s values, which focused on ensuring equitable access to quality health care through community and school involvement, conflicted with the existing government’s policies
Emergence over time		
Need for ongoing adaptation	*“There is uncertainty regarding the technology’s adaptation over time, as what is considered ‘relevant’ today may require ‘reinvention’ tomorrow.”* [Participant ID 6]	“Cadê o Kauê?’ needs to adapt to shifting technology policies, educational practices and funding priorities

a NASSS: Non-adoption, Abandonment, Scale-up, Spread, and Sustainability.

### Complexity Assessment

As illustrated in Table 2, the identified subthemes were mapped onto the NASSS domains. The findings were subsequently applied to the Complexity Assessment Tool, which revealed significant areas of complexity within the domains of technology, organization, and wider system. These were characterized by uncertainties, interdependencies, and unintended consequences (Table 3). Instead of categorizing NASSS domains as simple, complicated, or complex, the complexity assessment focused on examining how different forms of complexity unfolded during the implementation. This approach also acknowledges the dynamic nature of complexity, recognizing that a domain that is considered simple or complicated at one stage may evolve into a complex one over time, and vice versa.

**Table 3.** Table of complexities.

NASSS^a^ domain	Interdependencies	Unintended consequences	Uncertainties
Technology	Complex technical integrations require compatibility across multiple platforms. Smooth functioning is dependent on stable internet access and adequate infrastructure.	Provision of 3G routers during initial implementation enabled access, but students shared passwords for social media use, causing system crashes.	Teachers’ knowledge, beliefs, and attitudes towards technology still remain uncertain.
Organization	Adoption is contingent on organizational culture, readiness to innovate, and supportive leadership.	Implementation disrupted established routines, intervention delivery took longer than anticipated and teachers required additional training to both use the intervention and lead discussion circles effectively.	Uncertainty remains around the conditions required for routinization, as well as *who* should be responsible for introducing and mainstreaming the technology.
Wider System	Sustainability depends on political support, policy alignment, and system-level backing for spread and scale-up.	Polarized political climate and introduction of a new high-school system created disorganization in the initial implementation.	Fragmented technology policy landscape raises uncertainty regarding long-term sociopolitical support for sustaining the intervention.

a NASSS: Non-adoption, Abandonment, Scale-up, Spread, and Sustainability.

### Complexity in Technology

The initial implementation of “Cadê o Kauê?” revealed complexity in its current state of materiality, as its smooth functioning depends on internet connectivity. While the selection criteria for the pilot implementation included schools with access to computers, some schools either lacked internet connectivity or faced frequent disruptions.

Using “Cadê o Kauê?” through the website also caused stability issues due to its dependency on the internet, particularly when the internet signal was interrupted, resulting in the loss of interaction history.

*If you are using the intervention through the website and the signal is cut, you would lose the history of your interaction, so you would have to start all over again. This made it difficult for students to play the whole game without losing interest.*

[Participant ID 3]

A teacher from a rural school noted*,*

*Due to the school’s rural location, the internet signal was weak, and the students preferred to access the tool on their mobile phones.*

[Participant ID 8]

This dependence also resulted in unintended consequences, such as in one school, where students shared router passwords, causing network crashes, thereby disrupting the effectiveness of the intervention.

*At one school, we provided a router, and the students ended up sharing the password with other students to check social media on their phones. Soon, the entire school started using the router, which was meant for 40 and not 700, leading to a system crash*.

[Participant ID 1]

Participants observed that these dynamics not only exposed structural challenges related to technology but also generated some knowledge about the digital behaviors of young people.

*We discovered the value of the internet among young people, especially when students do not have it and want to check their social media apps on their phones.*

[Participant ID 2]

In some schools, some teachers were resistant to the use of technology via social media platforms. Their concerns primarily stemmed from negative perceptions about social media and worries that it would distract students from their learning. However, a participant noted that collaboration and the process of coproduction can help overcome these fears.

*There was an initial hesitation among teachers to join the project; however, through the process of co-production and forming a teacher advisory committee, some teachers eventually became strong advocates and ran campaigns in their schools to promote the intervention.*

[Participant ID 6]

There was a shared recognition among participants that intervening offline would reduce this dependency on the internet.

*From this experience, we concluded that an offline intervention would be more reliable given the current infrastructure. An offline version, similar to older software games, would work better than relying on an online connection*.

[Participant ID 2]

### Complexity in Organization

In this context, the organization refers to schools, and their readiness to adopt the technology was influenced by several factors. Key factors that emerged during the initial implementation included the schools’ capacity for innovation, ability to absorb new knowledge, overall implementation climate, and the strength of leadership.

“Cadê o Kauê?” was piloted in 7 state schools in Brasilia, and typically, the school committees recommended a teacher from the *Life Project* course to introduce the intervention within classrooms. The *Life Project* is a course proposed in the new high school curriculum in Brazil, designed to promote students’ self-awareness and autonomy, as well as a purposeful approach to life. This course aligned well with the objectives of the Engadajamente project. “Cadê o Kauê?” was designed to fit within regular class hours without adding extra work for teachers; however, in practice, the whole implementation process took 3-4 hours.

*Ideally, incorporating the tool and discussion circle into curriculum… should mean no extra work for teachers… but the whole process took longer than anticipated and this disrupted the normal class routines.*

[Participant ID 5]

Apart from technical difficulties, teachers attributed adoption uncertainties to transitional and communication issues associated with the recently introduced high-school system. They observed that the intervention might be more effective after the school’s curriculum was fully restructured.

*I believe the project could be carried out again now that the school curriculum is more organised and we have adapted to the new high school system, even though further changes are expected. Things are less fragmented within the school now for better adoption of the intervention.*

[Participant ID 9]

Teachers also emphasized the need for broader engagement to address the structural challenges, especially from the school’s leadership.

*Expanded coordination with school managers is very important…the administrators must see value in prioritizing these projects; otherwise, they will not put all their efforts into this project’s implementation*.

[Participant ID 6]

The intervention was also tested in a military school setting, which posed unique challenges due to its authoritarian structure and rigid leadership, contrasting sharply with the participatory environment typically required for youth mental health initiatives. However, the teacher representing this school challenged these constraints by implementing “Cadê o Kauê?” in her classroom.

Participants also highlighted teachers’ requests for additional training and the schools’ need for mental health resources. Teachers acknowledged that they often encounter mental health concerns among students, but their training typically lacks mental health literacy on handling such issues.

*This request for further training is not a minor adjustment; it suggests a need for more comprehensive training*.

[Participant ID 2]

*It is also important to provide more training and didactic support to teachers, as they can take valuable lessons from this project on how to handle difficult situations in classrooms concerning student mental health.*

[Participant ID 10]

### Complexity in External or Wider System

The co-design and implementation phase of “Cadê o Kauê?” were affected by the challenging sociopolitical climate, the introduction of a new high-school system, and fragmented social policies in Brazil. The project’s values, which focused on community participatory approaches in the school involvement, conflicted with the government’s policies. The ruling government did not prioritize the rights of vulnerable populations, including the mental health care needs of young people.

*Brazil faced significant challenges over the last few years, including economic decline, political instability, and a major shift towards extreme right-wing policies. The political climate created uncertainty around acceptance of educational interventions concerning adolescent mental health.*

[Participant ID 5]

During the initial implementation of “Cadê o Kauê?,” Brazil’s educational system also underwent significant restructuring with the introduction of a new high-school curriculum. This reform coincided with rising school dropout rates, sparking criticism that the reform was contributing to the decline in quality of Brazil’s education system.

Despite these challenges, the Engadajamente team navigated this complexity in the sociopolitical landscape. They were cautious about the design of the chat-story as a youth collaborator noted,

*We did not omit any mental health topics, but we did have to cut certain content due to political concerns. I suggested to tone down discussions on LGBTQ+ issues. A previous incident influenced this decision, in 2013, when an infographic addressing homophobia caused significant controversy and was not accepted in schools. I recommended taking a vigilant approach to avoid risking our access to schools*

[Participant ID 1]

## Discussion

### Principal Findings

Using the NASSS framework as a sensemaking device, this study captured diverse perspectives of stakeholders to generate a narrative of the initial implementation of “Cadê o Kauê?” in school settings in Brazil. By mapping the complexities across the NASSS domains and conducting a complexity assessment, we shed light on the dynamic relationships between technology and the broader social system around it. Building on these insights, we propose recommendations for addressing current and emerging complexities that may evolve over time.

### Managing Complexity in Technology

Access to the internet and technological infrastructure are prerequisites for most eHealth innovations [37], and these requirements could limit technology’s adaptability in resource-constrained settings [38]. Using a sociotechnical lens, 2 challenges are identified that may emerge over time due to this reliance on the internet. First, building on the views of Meyer et al [38], that while end users may have an overall positive view of “Cadê o Kauê?” the trust in its overall use may fade over time if users experience more than occasional failures and frustrations. Second, it could hinder the scale-up of intervention in schools with poor internet access and/or appropriate infrastructure. In turn, this dependency may widen existing inequalities, as schools without adequate infrastructure may be excluded from the benefits of this intervention. This digital divide is a concern among bioethicists, as digital mental health interventions may exacerbate existing inequities [35].

The first step to address complexities in this domain is to move away from viewing technology as a “plug-and-play” solution. To reduce the reliance of technology on the internet, one potential solution identified is to develop an offline version of the intervention; this approach could help maintain user engagement while also addressing the issues of equity. However, dependency on computers remains a concern if some are nonfunctioning. This factor largely depends on the organization’s capacity and willingness to support the innovation.

Knowledge, perceptions, and attitudes about the technology also shape its acceptance [37], so it is essential to thoroughly understand these dynamics early and integrate strategies to address them in project planning. In some schools, teachers were initially resistant to using the technology, particularly because it was delivered through social media platforms. Their concerns largely stemmed from negative perceptions of social media and fears that it could distract students from learning. However, these reservations were gradually addressed through collaborative approaches. Participation in coproduction processes and teacher advisory committees helped build trust. Over time, some teachers became strong advocates who actively promoted the intervention within their schools.

### Maximizing Value

The Engadajamente team members envision a future for “Cadê o Kauê?” where the intervention can be scaled nationally and embedded in the school curricula. However, lack of sustainable funding is a potential barrier to scale-up. Making a plausible business case for “Cadê o Kauê?” to generate long-term and alternative funding streams is challenging, owing to uncertainties in the redistribution costs over time [39].

The value of technology in a health policy context lies in the nature and scale of the benefits it generates [36]. To navigate the complex external environment, value-generation strategies are proposed to reframe the intervention’s benefits in line with policy priorities, enhancing its appeal to alternative investors and government stakeholders.

First, the intervention can be repositioned as a strategy to improve adolescents’ access to Brazil’s public mental health services. Although understanding care-seeking pathways to mental health care services is complex, nonetheless, there is a substantial unmet need in Brazil, with only 20% of children and adolescents using public mental health services [40]. Adolescents consulting primary care centers reported solving mental health problems generally on their own or with peers [41]. One component of “Cadê o Kauê?” directly addresses this gap by providing an information toolkit that maps free mental health services available through Brazil’s Unified Health System (Sistema Único de Saúde) across different states. This approach raises adolescents’ awareness of their right to access these services and encourages them to seek professional care for their mental health issues. This rights-based approach aligns with the principles of Brazil’s community mental health network (RAPS) [42]. A youth collaborator in the Engadajamente project with lived experience of mental health issues reflects how, as a teenager, they were unaware of such services and how having that information would have made a significant difference for them. Additionally, using schools as a platform to recognize early signs of mental distress, facilitate early detection, and refer cases to mental health care has been highlighted as a promising approach to improving care-seeking pathways [43]. Integrating “Cadê o Kauê?” into the school environment positions it as a bridge linking adolescents to existing public health infrastructure. This process aligns the intervention with national policies’ planned efforts to strengthen adolescent mental health care within Sistema Único de Saúde.

### Recognizing the Organization’s Readiness

Organizational readiness for change is considered a key factor in determining whether evidence-supported interventions are successfully adopted in a system [44]. Contextual factors, such as leadership, teamwork, trust, implementation climate, and resource availability, also shape implementation outcomes [44]. Drawing upon some of these factors, we explain how these link to the implementation of “Cadê o Kauê?” in schools. The school system is considered an inherently complex setting [45] where multiple components are interconnected and interact dynamically, and any successful intervention must account for and adapt to such complexity [46]. A “whole school approach” [47] that promotes collaborative efforts among administrative staff, teachers, and students, offers a promising strategy for implementation in schools as it addresses school-system complexities [47] and mobilizes collective action [48] across stakeholder groups.

It is, therefore, also essential to clarify role-specific responsibilities, particularly regarding *who* is responsible for introducing the innovation in the school and *who* drives it forward. Teachers are the primary facilitators of “Cadê o Kauê?” within classrooms and are responsible for conducting discussion circles afterwards. The complexity in pilot implementation in schools emerged from the need for additional training to support facilitation, as some teachers felt unprepared to address complex concerns around mental health that surfaced during the discussion circles.

Unfamiliarity and skepticism about innovation can also create initial hurdles in its uptake and sustainability [45]. Although a teacher advisory committee was constituted and consulted with as a part of the coproduction process, teachers must also be consulted about their willingness, knowledge, and beliefs about the technology and broader meaning of mental health through dedicated research. Engaging in collective sensemaking [31] with teachers from the outset, coupled with the provision of additional training, can help mitigate initial resistance toward the technology. Additionally, aligning the intervention with the school’s values and fostering mutual understanding through involvement of school administrators in the coproduction process can facilitate the transition and promote a supportive culture for innovation.

### Running With “Sociopolitical” and “Emerging” Complexity

During the COVID-19 pandemic, Brazil’s sociopolitical climate faced significant challenges [49], which interfered with the planning, design, and implementation of “Cadê o Kauê?” These tensions were faced due to political polarizations complicating the acceptance of adolescent mental health interventions. Similarly, conservative religious influences created resistance, especially concerning LGBTQ+ rights, leading to the cautious design of characters in the story “Cadê o Kauê?”

As we conducted the interviews with the Engadajamente team, participants shared coherent views and acknowledged inherent complexities in Brazil’s technology and educational policy landscape, especially with the introduction of technology regulatory policies and a new high school system. Until now, the team has navigated this uncertainty by “running” with it despite several roadblocks. We drew upon the values-informed approach to complexity by Greenhalgh et al [50], where engaging with such uncertainty requires human qualities like courage, humility, and flexibility. Researchers and youth collaborators from the Engadajamente team possess these qualities and have contributed to these human actions in navigating the complexity of the external environment. For instance, a youth collaborator showed “flexibility” when they recommended toning down the “language” and “certain characters” used in the “Cadê o Kauê?” story design to fit the complexities in the sociopolitical landscape. The policy expert embodied “humility” when she acknowledged that uncertainty in Brazil’s unripe technology policy landscape may be too early to sustain intervention like “Cadê o Kauê?” in its current version. The teacher in a military school exhibited “courage” by resisting the conflicting authoritarian values of the school and introduced “Cadê o Kauê?” within classrooms in the initial testing study.

### Strengths and Limitations

This study represents the first service evaluation project conducted by the Engajadamente team to assess the implementation of “Cadê o Kauê?” in schools. The evaluation design is strengthened by considering the broader social context of implementation as opposed to previous user testing studies conducted in isolation. The knowledge produced from this narrative will provide valuable insights to the Engajadamente team in identifying their priorities for further implementation. This evaluation is timely as the Engajadamente project has recently secured funding from the UK Economic and Social Research Council for further development and evaluation of “Cadê o Kauê?” which will enable the team to address some of the complexities identified in the study.

This study is not without limitations. Data analysis was conducted by SS, a non-Brazilian researcher, using translated transcripts. To minimize potential misunderstandings, she dedicated significant time to understanding the sociocultural context of Brazil, the transcripts were thoroughly checked for accuracy, and GP and SGM provided substantial input to support both data analysis and interpretation. Another limitation is the retrospective nature of participants’ reflections on the implementation. Future research conducted concurrently with the implementation phase could offer additional insights. Finally, the study could have been strengthened by diversifying the participant sample. Including more teachers and students involved in intervention testing in schools would have enabled a more comprehensive understanding of their diverse experiences.

### Translation to Policy and Practice

While this study was situated within Brazilian public school contexts, several findings may be transferable to other LMICs and school settings where digital mental health interventions are introduced into complex and resource-constrained systems. In particular, challenges related to organizational readiness, infrastructure variability, and alignment with existing institutional priorities are likely to resonate across similar educational and policy environments.

The application of NASSS–Complexity Assessment Tool in this study also demonstrates its utility for examining sociotechnical evaluation of technology implementation in LMIC contexts. Given that the framework has been used only in a small number of LMIC studies to date, our findings add to the emerging evidence based on its applicability for examining the sociotechnical complexity of technology implementation.

### Conclusion

Using the NASSS framework, this evaluation project has generated a rich narrative that captures both (1) the retrospective complexities encountered during the initial implementation of “Cadê o Kauê?” in schools and (2) the emerging complexities that may unfold with time, affecting its future implementation. By engaging with theory-informed approaches to technology implementation projects and deep-diving into discursive dynamic interactions between technology and its “context” of implementation, we have explored ways to manage these complexities and developed suitable recommendations to guide the Engadajamente team. The implementation of “Cadê o Kauê?” highlighted challenges related to internet dependency, infrastructure limitations, and varying levels of organizational readiness, teacher preparedness, and school culture factors that influence its successful integration and scalability. Addressing these through offline solutions, additional teacher training, whole-school engagement strategies, and a value-informed complexity approach can enhance accessibility, alignment with school priorities, and adaptability within Brazil’s dynamic socio-political context.

## Supplementary material

10.2196/79106 Multimedia Appendix 1 Semistructured Interview Guide.

10.2196/79106 Multimedia Appendix 2 Participation Information Sheet.

10.2196/79106 Checklist 1 COREQ checklist.

## Abbreviations

LMIC: low- and middle- income country
NASSS: Non-adoption, Abandonment, Scale-up, Spread, Sustainability
