# Supplementary material for: Using the NASSS–Complexity Assessment Tool to Evaluate the Implementation of “Cadê O Kauê?”: Chat-Story Intervention for Youth Participation in Mental Health Promotion in Brazil
Source: J Med Internet Res. 2026 May 27;28:e79106. doi: 10.2196/79106 (PMC13215631; doi:10.2196/79106)
Supplement: Multimedia Appendix 1 [file jmir-v28-e79106-s001.docx]

**Semistructured Interview Guide**

**This guide aims to capture diverse perspectives and experiences from stakeholders involved in the co-design and implementation of “Cadê o Kauê?”**

Table 1: Sample of the semi-structured interview guide

| Section 1: Introduction: Personal Background, Context and Technology   - Briefly describe your background and provide an overview of your involvement in the co-design and implementation of “Cadê o Kauê?” - Could you describe the focus of this tool? Do you feel this needed for Brazilian adolescents? - What was the Brazilian political context during implementation? Did that play a role in implementation? |
| --- |
| Section 2: Implementation Process   - Can you clarify your role in the implementation process? What were your responsibilities during this phase - How was “Cadê o Kauê” introduced to students? - Did you undergo training to run the implementation process? - Discuss any positive aspects, successes, or highlights. Are there any specific aspects of the tool that users find particularly effective or challenging? |
| Section III: Reflection and Future Directions   - Looking back, what aspects of the “Cadê o Kauê” implementation do you consider particularly successful? - Do you have any recommendations for improving the implementation of “Cadê o Kauê” in the future? - In your experience, what are the key factors for scaling up “Cadê o Kauê” at a national level? |

Table 2: Sample interview questions adapted to fit ‘Technology’ domain to be answered by Tech-Expert

| - Share your overall experience in developing “Cadê o Kauê” - Who are the primary users of the technology? - What are the key features and functions of “Cadê o Kauê” - Is the tool free to use? What is the cost of maintaining the tool? - Are there any privacy concerns? - Did you have to undergo any regulatory approvals? - What adjustments or modifications would you suggest for a more effective intervention? - In your experience, what are the key considerations for scaling up successful interventions like Cadê o Kauê? - Discuss any positive aspects, successes, or highlights. Are there any specific aspects of the tool that users find particularly effective or challenging? |
| --- |

Table 3: Sample interview questions adapted to fit the ‘Organisation’ domain to be answered by teachers

| - **Describe your background briefly** - **How was the** “Cadê o Kauê” **intervention received at your school?** - **Can you describe your role in the implementation of** “Cadê o Kauê” **at your school? Did you undergo any training?** - **Which aspects of the** “Cadê o Kauê” **implementation do you consider successful?** - **Can you share the challenges encountered during the implementation of the tool in schools?** - **What are the barriers and facilitators to the current adoption of** “Cadê o Kauê” **"?** - **Based on the challenges faced and the insights gained, do you have any recommendations for improving the implementation of** “Cadê o Kauê”  **in the future?** |
| --- |

Table 4: Sample interview questions adapted to fit the ‘wider context’ domain to be answered by policy expert

| - **Describe your background briefly** - What are the existing policies related to educational technology/tools in the schools? - How do you perceive the current educational landscape in Brazil regarding mental health interventions? - Are there specific socio-cultural or regulatory aspects that influence the implementation of digital mental health intervention in schools? - Are specific challenges or opportunities within the policy landscape affecting Cadê o Kauê - How do you envision the future of this tool in schools? |
| --- |
