# Supplementary material for: Using the NASSS–Complexity Assessment Tool to Evaluate the Implementation of “Cadê O Kauê?”: Chat-Story Intervention for Youth Participation in Mental Health Promotion in Brazil
Source: J Med Internet Res. 2026 May 27;28:e79106. doi: 10.2196/79106 (PMC13215631; doi:10.2196/79106)
Supplement: Multimedia Appendix 2 [file jmir-v28-e79106-s002.pdf]

Ethical Considerations

Consent will be taken from individual members of the Engadajamente team using a Microsoft Form (linked provided on email body). Using a participation information sheet, individuals will be duly informed about the nature of the project, objectives, procedures for data collection, confidentiality in the use of data, risks and benefits, voluntary nature of participation and right to withdrawal without any penalty. Individuals will have access to my contact details if they have any questions through the information sheet.

Data processing

Consent will be collected via a Microsoft Form, with a copy saved in a dedicated folder on the University OneDrive. Audio recordings will be made using an encrypted device. They will be uploaded to the University OneDrive as soon as possible and deleted from the device. Transcripts will be made from the recordings using the Teams embedded transcription service, anonymised and stored as Word documents on the University OneDrive. Team members interviewed will be assigned a code, which will be used to label their interview transcript. I will keep an excel file containing team members' codes, names and emails on OneDrive. The linkage file will be stored on the University OneDrive and both the linkage file, and the audio recording will be destroyed following data analysis. Only my supervisors and I will have access to identifiable information, with access granted to the MS IDREC for the purposes of monitoring and/or audit. The transcripts (anonymised) will be stored for at least 3 years after final publication or public release.

Risks to Individuals

Data collection centred on the team members' experience of implementation and adoption processes presents minimal risk. They will be invited to speak about their experiential learnings in utilising the tool and will not trigger any emotional discomfort. The interview facilitator/translator from the co-design team will be prepared to communicate with individuals so that they feel comfortable and safe sharing their ideas.

Benefits to Individuals

Online interviews will provide individuals a platform to reflect on their experiences in implementation of the program. This evaluation through the NASSS-CAT tool will generate recommendations to further help manage the complexities with the adoption. Financial or material benefits will not be offered to individuals.

Risk and Benefits for community

No risks to the wider community are anticipated. The results of this study will be disseminated in the form of articles and other visual resources to help advance interventions and policies to support collective action for youth mental health.

Participation Information Sheet

You are being invited to take part in a service evaluation project. Before you decide whether to take part, it is important for you to understand why this evaluation is being conducted and what it will involve.

Please take the time to read the following information carefully and discuss it with others if you wish. If there is anything which is unclear, or you would like further information, please contact me. Having done this, decide if you wish to get involved.

1. Why is the evaluation being conducted?

The objective of this service evaluation is to better understand the implementation and adoption process of "Cadê o Kauê" in schools in Brazil.

2. Why have I been invited to take part?

You are invited for an interview because you are part of the team responsible for the co-design and/or implementation of the "Cadê o Kauê" in schools.

3. Do I have to take part?

No. It is up to you to decide whether to take part. You can withdraw yourself from taking part, without giving a reason, and without negative consequences, by advising me of this decision. If you wish to withdraw your data from the study, you can contact me and I will delete your records immediately (however, please note that once I have transcribed and removed identifying information from the interview data, this will no longer be possible).

4. What will happen to me if I take part in the service evaluation?

- Consent will be taken using a Microsoft Form (linked provided on email body)
- You will then participate in an online interview (around an hour) via Microsoft Teams
- The interview is divided into three main stages
    - Stage I will cover questions about the Brazilian context and the technology itself.
    - Stage II moves on the implementation and adoption processes of the technology and the challenges related to it
    - Stage III lets you reflect on recommendations
- With your consent, we would like to audio record you so that we have an accurate record of our conversation that can be transcribed.
- You can pause or stop the interview at any point, and you can skip any question.

5. What information will be collected and why is the collection of this information relevant for achieving the objectives of the evaluation?

We will collect, process and share the following data to be able to conduct and analyse data from interviews:

- Interview recordings: Kept securely in university drives restricted to study team; deleted when data analysis is complete. Recordings might be transcribed by an external transcription company approved by the University of Oxford.
- Consent records: Kept securely in university drives restricted to study team; deleted 3 years

after final publication of the work.

- Interview transcripts: Following transcription, any information that might identify you or your organisation will be removed, and the file will be labelled using a random ID. Transcripts may be uploaded to data repositories or shared with other researchers via OneDrive. They will be kept for at least 3 years.
- Name and contact details: We will keep a file linking your ID to your name and in case we need to recontact you for further information. This file will be deleted following data analysis.
- All data collected during the evaluation may be looked at by authorised people outside the evaluation team, including the University of Oxford, for auditing and/or monitoring purposes.

6. Data Protection

The University of Oxford is the data controller with respect to your personal data, and as such will determine how your personal data is used. The University will process your personal data for the purpose of the evaluation outlined above. Further information about your rights with respect to your personal data is available from the University's Information Compliance website at https://compliance.admin.ox.ac.uk/individual-rights.

7. Are there any potential risks?

Data collection centred on the team members' experience of implementation and adoption processes presents minimal risk. They will be invited to speak about their experiential learnings in utilising the tool and will not trigger any emotional discomfort. The interview facilitator/translator from the co-design team will be prepared to communicate with individuals so that they feel comfortable and safe sharing their ideas.

8. Are there any potential benefits?

Online interviews will provide individuals a platform to reflect on their experiences in implementation of the program. This evaluation through the NASSS-CAT tool will generate recommendations to further help manage the complexities with the adoption. Financial or material benefits will not be offered to individuals.

9. What are the potential risks and benefits for the community?

No risks to the wider community are anticipated. The results of this study will be disseminated in the form of articles and other visual resources to help advance interventions and policies to support collective action for youth mental health.

10. Who has reviewed this project?

This is a service evaluation. As such, it has been considered exempt from ethics approval by the Oxford Tropical Research Ethics Committee. The University of Brasília Social Sciences and Humanities Ethics Committee. However, all the guidelines and principles from both universities guiding human research and data processing have been followed.

11. Who do I contact if I have a concern about the evaluation or I wish to complain?

If you have any concerns about any aspect of this project, please contact sakshi.setia@kellogg.ox.ac.uk and I will do my best to answer your query. We will acknowledge your concern and give you an indication of how it will be dealt with.

I confirm that I have read and understand the information above. I have had the opportunity to consider the information, ask questions and have had these answered satisfactorily.

I agree to take part in this service evaluation. (.).

Full name: ________________________________

What is your email?

What is/was your role in the Cadê o Kauê project?

[Please click here to download a copy of your answers for your records]
